# Maffucci syndrome and neoplasms: a case report and review of the literature

**DOI:** 10.1186/s13104-016-1913-x

**Published:** 2016-02-27

**Authors:** Olga Prokopchuk, Stephanie Andres, Karen Becker, Konstantin Holzapfel, Daniel Hartmann, Helmut Friess

**Affiliations:** Department of Surgery, Klinikum rechts der Isar, Technical University, Ismaningerstrasse 22, 81675 Munich, Germany; Institute of Human Genetics, Technical University, Munich, Germany; Institute of General Pathology and Pathological Anatomy, Technical University, Munich, Germany; Institute of Radiology, Technical University, Munich, Germany

**Keywords:** Maffucci syndrome, Cholangiocarcinoma, *IDH1/2*-mutations

## Abstract

**Background:**

Maffucci syndrome is characterized by the sporadic occurrence of multiple enchondromas together with multiple hemangiomas. Patients with Maffucci syndrome are at increased risk of developing different kinds of malignant tumors.

**Case presentation:**

We report on a 39-year-old woman who was diagnosed with Maffucci syndrome together with intrahepatic cholangiocarcinoma (IHCC). Heterozygous somatic mutations in the isocitrate dehydrogenase 1 and 2 (*IDH1/IDH2*) genes are associated with a number of different tumor types (e.g. IHCC) and also with Maffucci syndrome. For IHCC, mutations in *IDH1/IDH2* are associated with higher survival rates. IHCC tissue as well as normal liver tissue and peripheral blood were analyzed for *IDH1/IDH2*-mutations in our patient. In the tumor sample, we identified a recurrent somatic *IDH1*-mutation affecting Arg132, while in normal liver tissue and peripheral blood, no variants were detected, as expected.

**Conclusion:**

This case report presents the second patient in the literature exhibiting the features of Maffucci syndrome along with cholangiocarcinoma. This supports the hypothesis that *IDH1/2*-mutations, which can be present in different types of tumor tissue simultaneously, arise during embryonic development in a mosaic pattern; as a result, a more aggressive follow-up is proposed in patients with Maffucci syndrome to exclude neoplasms.

## Background

Maffucci syndrome (also known as dyschondrodysplasia with hemangiomas, enchondromatosis with multiple cavernous hemangiomas, Kast syndrome, hemangiomatosis chondrodystrophica, and enchondromatosis Spranger type II) was first described in 1881. It is a rare disease, with fewer than 200 cases having been reported worldwide to date. Maffucci syndrome is characterized by the presence of multiple enchondromas combined with multiple soft tissue hemangiomas or lymphangiomas [1]. There is a tendency for malignant transformation of enchondromas into chondrosarcomas or of hemangiomas into vascular sarcomas. Patients with Maffucci syndrome also are susceptible to the development of other malignant lesions such as glioma (summarized in Table 1).

**Table 1:** Description of Maffucci patients with or without neoplasms worldwide

Number of patients with Maffucci syndrome	Number of osteosarcoma cases	Number of non-skeletal neoplasms	Kind of non-skeletal neoplasms	Follow-up	Year of publication	References
1	1 probably malignant transformation of a clavicular lesion	0		No	1881	[11]
	0	3	Astrocytoma, pituitary adenomas, juvenile granulosa cell tumor		1973	[12]
7	4 (three of them had two neoplasms each)	3	BC, PC, astrocytoma	Yes	1987	[8]
1	0	1	Astrocytoma		1987	[13]
1	0	1	Astrocytoma		1990	[14]
1	0	1	Ovarian fibrosarcoma	No	1990	[15]
1	0	1	Astrocytoma		1991	[16]
1	0	0		No	2001	[17]
17	9/17 (53 %)	0		Yes	2011	[10]
1	1	1	Hemangioendothelioma	Yes	2012	[18]
1	1	0		No	2012	[19]
1	0	1	Spindle cell hemangioma	No	2013	[20]
1	1	0		No	2013	[11]
1	0	0		No	2013	[21]
1	0	1	Anaplastic astrocytoma	No	2014	[22]
1	1	0		No	2014	[23]
1	0	0		No	2014	[24]
1	1	0		No	2014	[25]
1	1	0		No	2014	[26]
9	1	1	Acute myeloid leukemia and von Willebrand disease	No	2014	[27]
1	1	0		No	2015	[28]
1	1	0		No	2015	[29]
1	1	0		No	2015	[30]
1	0	0		No	2015	[31]

*PC* pancreatic cancer, *BC* biliary adenocarcinoma

Somatic mutations in the isocitrate dehydrogenase 1 (*IDH1*) or *IDH2* genes are common in enchondromas and chondrosarcomas, as well as in several neoplasms, including glioma, glioblastoma, acute myeloid leukemia, and intrahepatic cholangiocarcinomas (IHCC) [2–4].

## Case presentation

A 39-year-old woman was referred to the department of surgery because of a large tumor in her right hepatic lobe. The liver node was found incidentally at sonography. The patient did not complain of any abdominal problems.

The patient’s medical history reported that at the age of 7 years, she had been diagnosed with two nodes on the heel suspicious of hemangiomas, and at the age of 16 years she developed diffuse nodes suspicious of hemangiomas. One subcutaneous hemangioma in the upper lumbar region had been surgically removed and histologically confirmed in 2000. From 2003, she had a history of multiple enchondromas on her right fibula, ribs and clavicle, and the diagnosis of Maffucci syndrome was therefore proposed. As an inhabitant of Kyiv, the patient had been exposed to radiation from Chernobyl as a child.

**Fig. 1:**
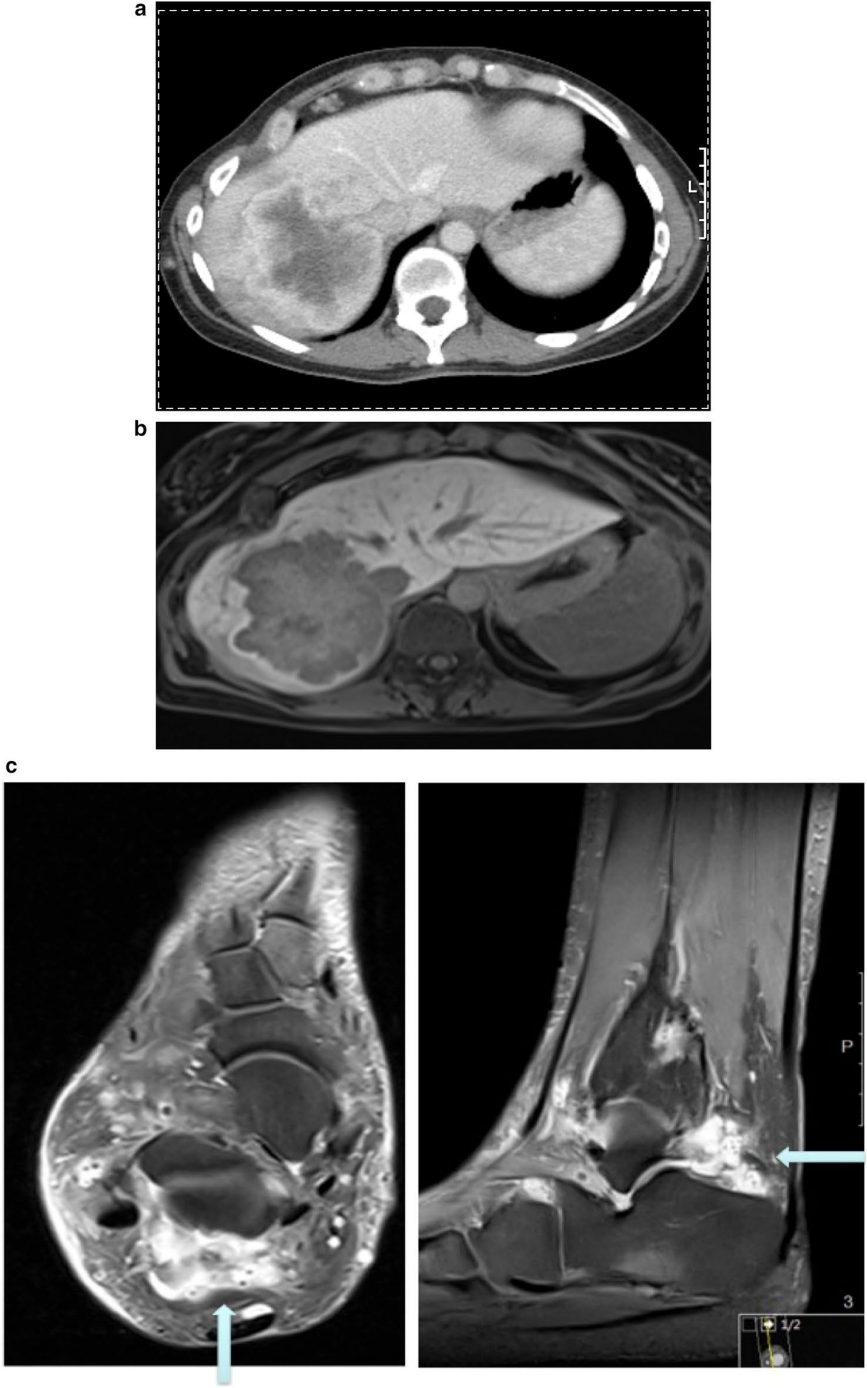
Preoperative imaging studies. **a** Computed tomography of liver tumor showed a 9 cm mass in the right lobe of liver; **b** Gadoxetic acid (primovist)-enhanced MRI demonstrated a lesion in liver segments VII and VIII, peripherally hypointense compared to surrounding *liver in T2w*, centrally hyperintense. A strong arterial hypervascular enhancement was demonstrated after primovist administration and there was a failure to enhance in the hepatobiliary phase. The tumor has contact with the inferior vena cava and some mm distance from middle liver vein. T1 fat saturated post gadoxetic acid. Hepatobiiliary phase (20 min post injection). **c** Soft tissue hemangioma (MRI). Hemangioma dorsal to upper ankle joint, PDw fat saturation (proton density weighted, fat saturated MRI)

Computer tomography (Fig. 1a) showed a mass in the right hepatic lobe measuring 9 cm in the largest dimension. Whereas ultrasound-guided biopsy did not achieve a definitive histological diagnosis (cytology showed suspicious cells), the tumor was highly suggestive of a neoplasm on computed tomography (CT) and magnetic resonance imaging (MRI). A failure to enhance on gadoxetic acid (primovist)-enhanced MRI (Fig. 1b) excluded adenoma and focal nodular hyperplasia (FNH). Primary staging, including gastroscopy and colonoscopy, did not reveal any other tumor sites. The preoperative differential diagnosis was primarily between hepatocellular carcinoma and sarcoma. Resection was recommended by our multidisciplinary tumor board. The patient underwent right extended hemihepatectomy (V + VI + VII + VIII + IVa segments) with cholecystectomy. Additionally, two subcutaneous hemangiomas were removed from the abdominal wall.

## Methods

### Pathology

Representative areas from the right hepatectomy specimen were sampled, and 5-μm sections from the formalin-fixed, paraffin-embedded tissue were used for routine light microscopic analysis, as well as immunohistological analysis. The antibodies used for immunohistology were HepPar, Synaptophysin, Chromogranin A, ERG, CKpan, S-100, CK7, CK20, CDX 2, CEA, CA19.9, PAX8, ER, PR, and CA12.5.

#### Histopathologic findings

**Fig. 2:**
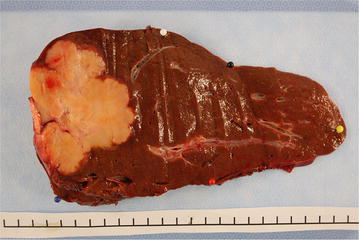
Pathological findings. Right hemihepatectomy tissue

**Fig. 3:**
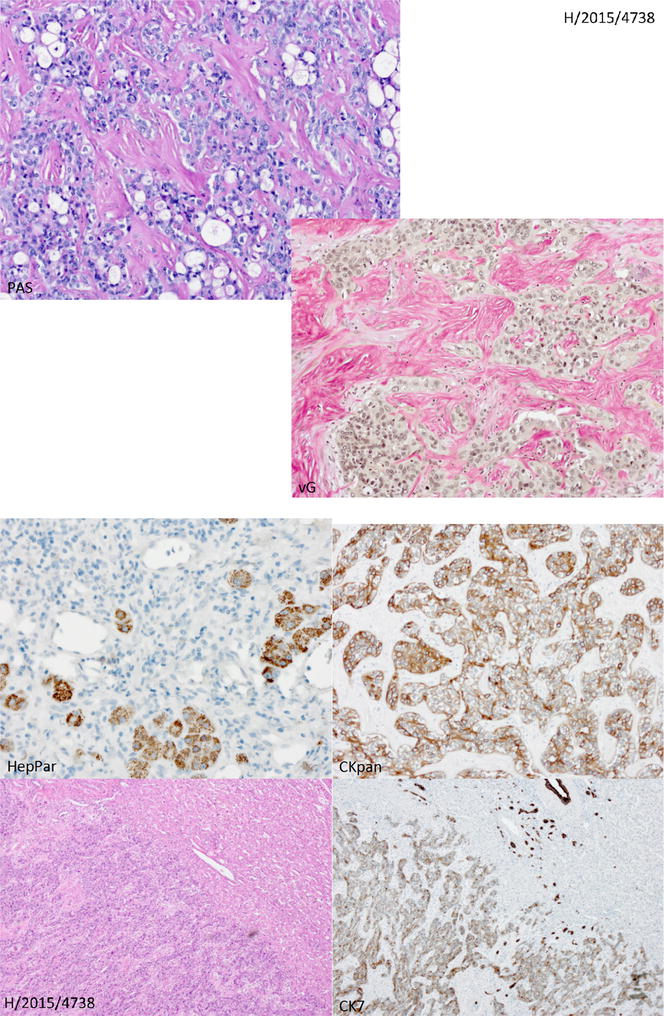
Histology and immunhistology of cholangocarcinoma

The right hemihepatectomy tissue had a weight of 725 g and dimensions of 21 × 12 × 7.5 cm. The tissue included part of the diaphragm 4 × 2 cm from the cranial side. The tumor was multinodular and 8.5 × 6.5 × 6 cm in size (Fig. 2). Histologically, it was liver tissue with infiltration of tubular to partially solidly growing adenocarcinoma, with middle proliferative activity and a focal hepatocellular component, which was a maximum of 8.5 cm. Immunohistochemical staining of neoplastic cells showed strong staining with cytokeratin 7 (CK7) and CPpan (Fig. 3), and weaker staining with cytokeratin 20 (CK20). Some tumor cells displayed weak staining for HepPar1 (Fig. 3). The tumor cells were negative for synaptophysin, chromogranin A, Ets-related gene (ERG), S-100, CDX 2, carcinoembryonic antigen (CEA), CA19.9, PAX8, estrogen receptor, progesterone receptors, cytokeratin 19 (CK19), CA19.9, GATA 3, and alpha-fetoprotein (AFP), as well as for CA12.5. There was a focal intracytoplasmic positivity in PAS-staining (Fig. 3). The proliferation rate, visualized with MIB1, was seen in hot spots, up to 10 %.

**Fig. 4:**
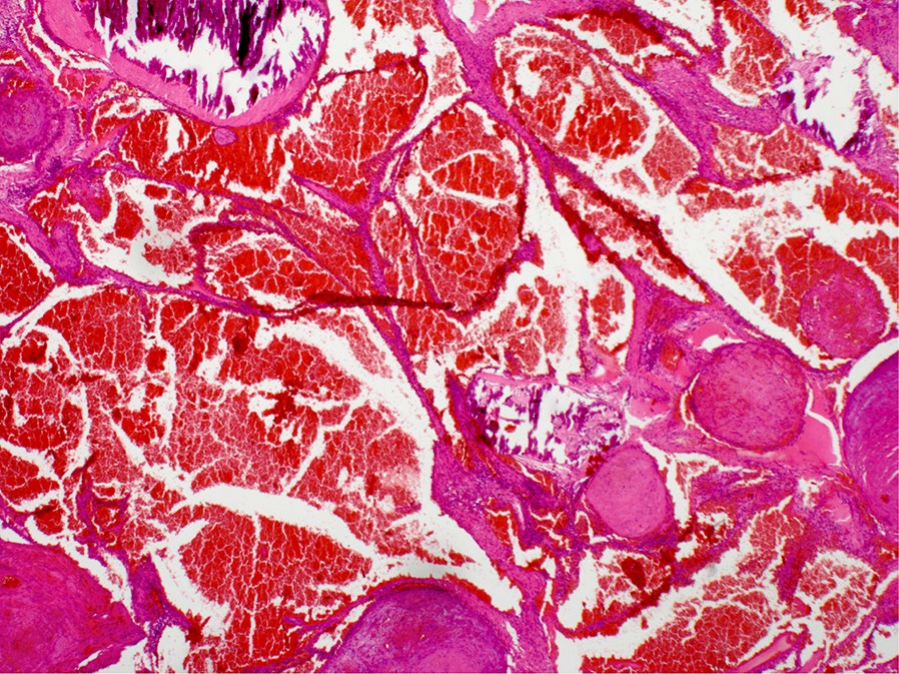
Histology of subcutaneous hemangioma

The adjacent non-tumorous liver tissue did not reveal any significant histopathological abnormality. The final diagnosis was stage I peripheral intrahepatic cholangiocarcinoma pT1, R0, G2.

In addition, two subcutaneous nodes were removed from the abdominal wall. Histologically, both of them were partially thrombosed capillary hemangiomas (Fig. 4).

### Genetics

DNA was extracted from paraffin-embedded tumor tissue, from paraffin-embedded normal liver tissue and from peripheral blood. We analyzed the 10 coding exons of the *IDH1* gene by Sanger sequencing and detected the mutation c.394C > T (p.Arg132Cys) in exon 4 of *IDH1* in the heterozygous state. This mutation leading to the amino acid change from arginine at position 132 to cysteine is a recurrent one and is found in the majority of the analyzed *IDH1*-positive tumor tissues [5]. In order to complete the genetic analysis, we subsequently Sanger-sequenced the *IDH2* gene with no remarkable results.

Biochemically, mutations in *IDH*1/*IDH*2 impair the substrate affinity of the enzyme and dominantly inhibit wildtype IDH1 activity through the formation of catalytically inactive heterodimers [6].

Heterozygous somatic mutations in isocitrate dehydrogenase 1 and 2 (*IDH1*/*IDH*2) are key events in the development of different kinds of malignant tumors, such as glioma, acute myeloid leukemia and intrahepatic cholangiocarcinoma (IHCC) [5]. They are also associated with Maffucci syndrome: over 80 % of patients with Maffucci syndrome carry somatic mutations in *IDH1/IDH2* [3], while *IDH1/IDH2* mutations are detected in 14 % of non-syndromic cases with IHCC [7].

## Discussion and conclusion

Patients with Maffucci syndrome can develop both skeletal and non-skeletal malignant lesions such as glioma [8]. As enchondromas and gliomas of Maffucci patients carry *IDH1*/*IDH*2 mutations while wild-type *IDH1*/*IDH*2 is expressed in their healthy tissue it is concluded that heterozygous *IDH1/IDH*2 mutations occur during embryonic development, leading to mosaicism and acting as triggers of carcinogenesis [5].

Our case strengthens this hypothesis due to the presence of a somatic *IDH1*-mutation in the IHCC tumor tissue of a patient with Maffucci syndrome. The presence of the mutation in enchondroma/hemangioma tissue of the patient remains to be confirmed.

The coexistence of Maffucci syndrome with cholangiocarcinoma is rare. To our knowledge, this case is the second to be reported. The first case was described in 1987. The female patient with Maffucci syndrome developed a chondrosarcoma of the femur at the age of 32 and a biliary adenocarcinoma at the age of 44, which she died from [8].

Our patient was exposed to the Chernobyl fall-out at the age of 11. It has been demonstrated that three types of liver cancer (hemangiosarcoma, cholangiocarcinoma and hepatocellular carcinoma) are significantly associated with chronic exposure to high LET α-particle radiation [9]. However, considering the clinical and genetic findings, this exposure to nuclear radiation did not appear to play a major role in this case.

For IHCC cases, mutations in *IDH1/IDH2* are associated with prolonged survival. The probability of tumor recurrence in patients with mutated *IDH1*/*IDH2* intrahepatic cholangiocarcinoma is significantly lower than in those with wild-type *IDH1/IDH2* (45 and 81 %, respectively, for a 7 year interval) [7]. So far, the reason for this difference is not fully understood.

An important question concerns the monitoring of patients with Maffucci syndrome for the early detection of malignancies. In order to identify chondrosarcomas, Vedegaal and co-workers proposed technetium scans in patients with more than one enchondroma. X-rays of every single enchondroma have been recommended to provide a baseline for future comparison [10]. To identify non-skeletal neoplasms, some authors have advocated a cerebral or abdominal CT when neurological or abdominal symptoms appear [10]. Our patient clearly demonstrates that the moment of clinical manifestation may be too late for successful management. In this case, an incidental sonography detected the IHCC, which is an aggressive neoplasm with an overall 5-year survival rate of 25–35 % after surgical treatment. For follow-up, we recommended a whole body MRI annually in addition to the usual oncological care. Future studies should aim to establish appropriate monitoring for patients with Maffucci syndrome to detect malignancies early.

## Consent

Informed consent to publish the information was granted by the patient.
